# Prevalence and Associated Factors of Anemia among Reproductive-Aged Women in Sayint Adjibar Town, Northeast Ethiopia: Community-Based Cross-Sectional Study

**DOI:** 10.1155/2020/8683946

**Published:** 2020-08-08

**Authors:** Berhanu Woldu, Bamlaku Enawgaw, Fikir Asrie, Elias Shiferaw, Zegeye Getaneh, Mulugeta Melku

**Affiliations:** Department of Hematology and Immunohematology, School of Biomedical and Laboratory Sciences, University of Gondar, Gondar, Ethiopia

## Abstract

**Background:**

Globally, anemia affects one-fourth of the world population including 30% of nonpregnant reproductive-aged women. It has a number of causes including micronutrient deficiencies and chronic infections, inherited or acquired disorders of hemoglobin synthesis and red blood cell production, or survival alterations. The aim of this study was to assess the prevalence and associated factors of anemia among reproductive-aged women in Sayint Adjibar town, South Wollo Zone, Northeast Ethiopia.

**Methods:**

A community-based cross-sectional study was conducted from February to April among 359 reproductive-aged women (RAW). Systematic random sampling technique was implemented to select study participants. Sociodemographic, socioeconomic, and reproductive histories of study participants were collected using the structured and pretested questionnaire. Capillary blood and stool samples were collected from each study participant for hemoglobin and parasitological analysis, respectively. Data were entered into Epi Info version 7 and transferred to SPSS version 20 for analysis. Both bivariable and multivariable binary logistic regression models were fitted to identify associated factors of anemia. *p* value <0.05 was considered as statistically significant.

**Result:**

The median age of the study participants was 25 years. The overall prevalence of anemia was 24.2%. Among those anemic individuals, 49 (56.3%) were mildly anemic. Age category 36–49 years (AOR = 2.64; 95% CI: 1.05, 6.60), no formal educational status (AOR = 2.28; 95% CI: 1.06, 4.92), food insecurity (AOR = 1.92; 95% CI: 1.01–3.65), and body mass index of above 25 kg/m^2^ (AOR = 0.27; 95% CI: 0.08–0.87) were found to be statistically significant with anemia.

**Conclusion:**

The prevalence of anemia in this study was found as a moderate public health problem. The prevalence was significantly associated with women who had no formal education and were of older age group and those women living with household food insecurity and with higher body mass index. Therefore, it is better to design appropriate interventional strategies to reduce reproductive-aged women anemia. These include information, education, and communication activities focused on reproductive-aged women with no formal education and life-cycle-focused food security rather than targeted to only infants and young children or pregnant women.

## 1. Background

Anemia is defined as a decrease in Red Blood Cells (RBCs), Hemoglobin (Hb), and hematocrit below the reference range for healthy individuals of the same age, sex, and race, under similar environmental conditions [1]. For nonpregnant women, it is defined when the Hb level is below 12 g/dl [2]. Anemia indicates a deficiency of one or more essential nutrients, heavy blood loss, hookworm infection, acute and chronic infections, and hemoglobinopathies [3]. Based on its public health problem, anemia can be categorized as follows: greater than 40%, 20–39%, and 5–19%, corresponding to severe, moderate, and mild public health problems, respectively [4]. Hb measurement plays a vital role in the diagnosis of anemia since it is inexpensive and easy to measure at field testing [3].

Anemia is a global problem affecting persons of all ages [5]. It results in reduced oxygen-carrying capacity of RBCs which will cause chronic fatigue and ill health [6]. Pallor, low blood pressure, headache, and edema are the most common manifestations of anemia. Moreover, general clinical findings may be characteristically associated with a specific type of anemia [7].

Globally, anemia affects 24.8% of the population [8]. Nonpregnant reproductive-aged women were the third most affected group next to preschool-aged children and pregnant women [9]. According to 2011 report, globally, the prevalence of anemia among nonpregnant reproductive-aged women was 29% with 1.1% of them being severely anemic. Report showed that the prevalence of anemia in East Africa was 28%, of which 1.2% were classified as severe anemia [10]. Based on the Ethiopian Demographic and Health Survey (EDHS) 2016 report, the prevalence of anemia among RAW in Ethiopia was 23% [11].

Anemia has a number of causes, with the most significant cause being iron deficiency. Approximately 50% of cases of anemia are considered to be caused by iron deficiency, but the proportion probably varies across population groups and in different geographical areas, based on the local conditions. In this regard, a systematic analysis of national surveys revealed that iron deficiency contributes to 37.0% of anemic cases. The other causes of anemia include other micronutrient deficiencies like folate, riboflavin, vitamins A and B12, acute and chronic infections and inherited or acquired disorders of hemoglobin synthesis, red blood cell production, or survival alterations [12, 13]. It is more common in women due to the loss of iron during menstruation, high demand throughout pregnancy, and puberty. Deprived nourishment, detrimental eating lifestyle, parasitic infestations, particularly malaria and hookworm, and rural residence are other contributing factors to the prevalence of anemia among women [14].

The World Health Organization targets a 50% reduction of anemia in RAW by the year 2025 [15]. Most of the studies conducted in Ethiopia were mainly focused on anemia of pregnant women even though RAW are also vulnerable. However, in Ethiopia, there are limited studies conducted to assess the prevalence and associated factors of anemia among RAW. Understanding the prevalence and associated factors will enable formulating effective interventions which are evidence based. So, this study aimed to assess the prevalence and associated factors of anemia among RAW at Sayint Adjibar town, South Wollo zone, Amhara region, Northeast Ethiopia.

## 2. Methods

### 2.1. Study Design and Period

A community-based cross-sectional study was conducted from February to April 2018 in Sayint Adjibar town.

### 2.2. Study Setting, Population, Sample Size, and Sampling Techniques

The study was conducted in Sayint Adjibar town, Amhara Sayint District, South Wollo Zone of Amhara Regional State. It is located between 12°13′N latitude and 38°53′E latitude covering a total area of 1,437.30 square kilometers. It is 189 km far from Dessie and 590 km from the capital city, Addis Ababa, Ethiopia, in Northeast direction. According to the 2011 sample survey data, Amhara Sayint district has a total population of 154,142, of whom 75,979 are men and 78,163 are women [16]. Based on the town municipality 2010 Ethiopian calendar demographic data, the town population size was 9693 which comprises 5306 females and 4387 males. There are a total of 2254 households in the town. The altitude of Sayint Adjibar town is 2885 meters above sea level, and the altitude of the Amhara Sayint District ranges from 500 meters to 3,700 meters above sea level. Selected and voluntary RAW who were living at Sayint Adjibar town during the study period were used as the study population.

The sample size was calculated using a single population proportion formula (*n*=(*Zα*/2)^2^ × *p*(1 − *p*)/*d*^2^) by considering a 30.4% prevalence of anemia among RAW [17], 5% margin of error, 95% confidence interval, and a 10% nonresponse rate. The final sample size then becomes 359. A systematic random sampling technique was used to select the study participants. Based on the size of the RAW and house number, the *K* value was calculated as 6. The first study participant (household) was selected randomly between 1st and 6th using the lottery method, and the next participants were selected in accordance with the 6th values until the calculated sample size was reached. A household with more than one RAW, one woman was randomly selected and included in the study. If there is no eligible woman within the selected household, the next house was selected.

### 2.3. Study Variables

The dependent variable was anemia status, and the independent variables were sociodemographic characteristics (age, marital status, education, household size, religion, and occupation), intestinal parasitic infections, socioeconomic status of the women (wealth index of the household and food security), the anthropometric measurement that is Body Mass Index (BMI), and reproductive health history of the women.

### 2.4. Data Collection Tools and Method of Data Collection

A pretested structured questionnaire was used to collect data about the study participants. Three data collectors (one clinical nurse and two laboratory technicians) participated in the data collection. The questionnaire was organized into different categories. The first part of section one includes the sociodemographic part of the study participants; section two dealt with the home environment like house nature. The second part contained questions about reproductive health-related questions, and the third part includes house food security status. The food security of the household was assessed by the response of seven questions with each response coded as no (0) and yes (1). Then, the possible food security of the household was calculated as the sum of the responses of the food security assessment questions, all responses coded “no” or 0, representing the most food-secure state and all responses coded “yes” or 1, representing the most food-insecure state. The scores were categorized as food secure, moderately food insecure, and severely food insecure [18]. The principal component analysis was used to derive a wealth index from information on ownership of the household assets. Principal components with eigenvalues greater than one retained to construct wealth index values and then categorized into three relative measures of the socioeconomic status of households as low, medium, and high.

### 2.5. Sociodemographic, Reproductive History, and Socioeconomic Data Collection

The sociodemographic data about the study participants were collected using a structured questionnaire which was adopted from the Ethiopian national micronutrient survey report as a data collection tool [19]. The questionnaire included sociodemographic characteristics like age, educational status, occupation, menstrual history including frequency and duration, contraceptive use, and number of pregnancies.

### 2.6. Anthropometric Measurements

Height and weight of the women were measured, and BMI was calculated. Weight was measured to the nearest 0.1 kg with an electronic digital scale. Each woman was weighed with minimum clothing and barefoot. The weighing scale was set to zero before every measurement and was calibrated using the standard calibration weight of 2 kg iron bars every day. For height and weight, individuals were requested to remove their shoes before taking measurements. Height was measured to the nearest 0.5 cm using a locally manufactured wooden stadiometer with a sliding headpiece. The women were instructed to stand straight with their heads, backs, and buttocks vertically aligned to the height gauge, and then their heights were taken and rounded to the nearest 0.5 cm. The BMI was calculated by taking the ratio of weight to height squared [weight (kg)]/[height in m^2^)] [20]. According to WHO, the standard cut-off value for underweight in RAW of nonpregnant women is a BMI of <18.5. BMI is normal when it is between 18.5 and 24.9, and it is considered overweight when it is greater than 25 kg/m^2^ [21].

## 3. Laboratory Examinations

### 3.1. Hemoglobin Determination

The Hb concentration of each participant was measured by taking a finger-prick blood sample using a portable hemoglobinometer instrument (HemoCue 301+, Ängelholm, Sweden) which is recommended by WHO for the use of field surveys [22] by using the procedure [23]. Then, Hb was corrected for altitude of town as proposed by the WHO [24].

### 3.2. Stool Sample Examination

The women were given a clean and leakproof container and kindly requested to bring a pea-sized stool sample. The samples were collected during the data collection day. Then, the stool sample was transported to Sayint health center and examined for intestinal parasites. Samples were preserved by 10% formalin, if not examined within 1 hour of collection. Wet mount examination method was employed to examine for the presence of intestinal parasites. Fresh stool sample was used for wet mount preparation, while on uncertain situations where we were unable to run immediately, the sample was preserved and examined later. Quality of preservative solution had been checked before being used.

### 3.3. Data Quality Management

In order to keep the consistency, the questionnaire was prepared in English and translated to Amharic then back to English to check for consistency. The data collectors were given training about the objective, relevance of the study, confidentiality of information, and study participants' rights before actual data collection. To standardize the questionnaire, pretest was conducted on 5% of the sample size outside of the study area among RAW before the actual data collection will take place. All laboratory reagents were checked for their expiry date. The quality control for HemoCue machine was carried out based on the manufacturer's instruction. The quality of the balance was checked by measuring known weight.

### 3.4. Data Processing and Analysis

The data entered into EPI Info 7 and checked and cleaned for completeness and consistency. Then, the data was transferred to a Statistical Package for the Social Sciences (SPSS) version 20 for analysis. Descriptive analysis like frequencies, percentages, means, medians, interquartile range (IQR), and standard deviations were calculated using the software. A bivariable binary logistic regression model was fitted to identify factors associated with anemia. Variables with a *p* value of <0.25 in the bivariable analysis were fitted into the multivariable binary logistic regression analysis to adjust the confounding factors. Both Crude Odds Ratio (COR) and Adjusted Odds Ratio (AOR) with the corresponding 95% Confidence Interval (CI) were calculated to show the strength of association. In the multivariable analysis, variables with a *p* value of <0.05 were considered statistically significant.

## 4. Results

### 4.1. Sociodemographic, Socioeconomic, and Reproductive Health-Related Characteristics of the Study Participants

The median (IQR) age of the study participants was 25 [18–32] years. From a total of 359 study participants, the majority—169 (47.1%)—of them were in the age group of 15–24 years. About 19% of the participants were living in households with a family size of greater than four members. Out of the total study participants, 87 (24.2%) were with no formal education, 210 (58.5%) had a secondary school and above education, and 128 (35.7%) were students (Table 1).

### 4.2. Prevalence and Associated Factors of Anemia among RAW

The overall prevalence of anemia among RAW in Sayint Adjibar town was found to be 87 (24.2%; 95% CI: 19.8, 28.7%). The Hb value of the study participants ranged from 8.1 g/dl to 15.7 g/dl after an adjustment to altitude. The median (IQR) Hb of the study participants was 12.7 (12.00–13.6 g/dl). Of the anemic study participants, 49 (46.3%) were found mildly anemic. However, there was no severe anemic case. Among anemic RAW, 23 (37.1%) were divorced and widowed and 27 (45.0%) were greater than 35 years old. Out of the anemic study participants, 37 (42.5%) had no formal education (Table 2).

To determine the association, bivariable and multivariable logistic regression analyses were done. Based on the analysis, variables with a *p* value less than 0.25 were included for multivariate logistic regression. Accordingly, age category, marital status, educational status, household food security, bleeding history, and the presence of intestinal parasites were significantly associated with anemia. However, in multivariable binary logistic regression analysis, age category (AOR = 2.64; 95% CI: 1.05, 6.60), educational status (AOR = 2.28; 95% CI: 1.06, 4.92), food security (AOR = 1.92; 95% CI: 1.01, 3.65), and BMI (AOR = 0.27; 95% CI: 0.08, 0.87) remained significantly associated with anemia with a *p* value less than 0.05 among RAW, but wealth index which might be related with the socioeconomic status and food security of the study participants was not significantly associated with the prevalence of anemia (Table 2).

We checked all 359 study participants' stool samples for intestinal parasites. From a total of RAW, 157 (43.7%) of the study participants had one of the seven intestinal parasites with a prevalence of *Ascaris lumbricoides* 82 (22.8%), *Entamoeba histolytica*/*dispar* 24 (6.7%), *Tania* species 15 (4.2%), *Giardia lamblia* 10 (2.8%), *Enterobius vermicularis* 9 (2.5%), *Trichuris trichiura* 9 (2.5%), and *Hymenolepis nana* 8 (2.2%). Of those women infected by intestinal parasites, 43 (27.7%) were found as anemic.

## 5. Discussion

The prevalence of anemia can be classified as severe, moderate, and mild public health problem when the prevalence is greater than 40%, 20–39%, and 5–19%, respectively. Based on the public health importance, our finding is categorized under moderate public health importance [4]. This indicated that a considerable number of women in the community were suffering from anemia. Anemia is negatively correlated with physical activity, leading to poor productivity and poor health outcomes. Therefore, emphasis should be given to reduce the prevalence of anemia in RAW. In 2011, WHO estimated that anemia among RAW in Ethiopia was a mild public health problem with a prevalence of 19%, of whom 1.1% were severely anemic [12]. However, in this study, there was no severe anemia detected.

In this study, the overall prevalence of anemia among RAW was found to be 24.2%. This result is consistent with the EDHS report of 2016 (23%) [11], a study conducted in Sidama zone of Southern Ethiopia (21.3%) [25]. On the other hand, it is lower than other studies conducted in Coimbatore district, India (64.4%) [26], in urban slums of Lucknow city, India (69%) [27], in Bursa, Turkey (32.8%) [28], Tanzania (39.6%) [29], Bangladesh (41.3%) [30], in urban slum of Mumbai, India (49.5%) [31], and Nepal city (37.6%) [18]. Moreover, the prevalence of anemia in this study was higher than those of the studies conducted in Jimma, Ethiopia (16%) [32], 2011's WHO report for Ethiopia (19%) [12], in rural Tabas, Iran 13.8% [33], and Vietnam 19.7% [34]. The possible reason for the variation between our findings with aforementioned studies might be due to the difference in the study period, socioeconomic difference, and geographical location of the study area above sea level.

In this study, educational status was found to be significantly associated with the prevalence of anemia among study participants. RAW with no formal education were more likely to be anemic as compared to RAW having secondary and above educational status. The odds of to be anemic in this study was 2.28 times higher in women with no formal education than women attending secondary and above educational status. This is supported by studies conducted in Ethiopia [32, 35], that low educational status was a key predisposing factor of women to be anemic. The reason might be due to the fact that the provision of education enables people to earn or increase their earnings so as to get out of the poverty trap. It is important to bring sustainable and effective poverty reduction [36]. In fact, anemia by its nature related to nutritional deficiencies, and in turn, nutritional deficiency has been directly related to poverty which leads to the onset of anemia. Education also directly impacts health and nutritional status [36].

On the other hand, wealth index was not significantly associated with prevalence of anemia. The possible reason might be due to the fact that the principal component analysis uses arbitrary classification of wealth in which the number of components and variables to include is not well defined and choice of the variables to assess the wealth index may be the other reason. The choice of variables included can have an impact on the observed poor-rich difference in health outcomes. The other possible reason may be inclusion of variables which had low frequency [37].

Age category of the study participants was also found to be significantly associated with the prevalence of anemia. This study revealed that being in the age category of 36 and above is 2.64 times more likely to be anemic as compared to the age categories of 15–24 years. The reason might be due to the economic burden of the women because most of the study participants in this age group were divorced and widowed. This result is consistent with the studies conducted in nine administrative regions, Ethiopia [17] and in Tabas, Iran [33]. The reason might be due to the fact that women under these age categories suffer from undernutrition [38]. This undernutrition, in turn, leads to the development of anemia. However, other studies in Jimma, Ethiopia, and in an urban slum of Mumbai, India, showed that age category between 25 and 35 had a greater risk of developing anemia than other age groups [31, 32]. Age category also significantly associated with anemia in a study conducted in Nepal city, but they found that being in the young age category was a risk factor for anemia [18]. The possible reason for the increment of the risk of anemia in the age group of 36 and above may be related to reproductive history, pregnancy history-related conditions, and maternal workload that may contribute to the high prevalence of anemia among those age groups [17].

This study identified that household food insecurity level has been significantly associated with anemia. Women living in food-insecured household were 1.92 times at higher risk of developing anemia as compared to women living in food-secured household. This result is supported by a study conducted in Mexico showing that household food insecurity among adult women was significantly associated with anemia [39]. It is comparative with a study conducted in Bangladesh on the association between food insecurity and anemia among women of reproductive age. There was an association between food insecurity and prevalence of anemia [40]. Poor maternal nutrition is directly associated with mother's lack of resistance to infection and with maternal ill health during pregnancy and childbirth [41]. This vulnerability for infections leads to the development of anemia. The possible explanation for the association between food insecurity and anemia is that as mentioned earlier anemia is mainly related to nutritional deficiency. Food insecurity had a substantially higher risk of suffering from anemia as compared to their food-secure counterparts. Food insecurity can lead to anemia through the inadequate intake of micronutrients [40].

In our study, we did not assess the iron status of the study participants. Even though it was not assessed, the iron deficiency may be related to the food insecurity. The food insecurity directly results in the lack of necessary nutrients including iron deficiency. The iron deficiency in turn results in the development of iron deficiency anemia, in which the iron deficiency anemia contributes to the prevalence of anemia. Fortification of suitable food vehicles with absorbable forms of iron is a highly desirable approach to controlling iron deficiency. This control mechanism of iron deficiency is directly related to food security [40, 42].

In this study, prevalence of anemia among RAW was significantly associated with BMI. Being of BMI greater than 25 kg/m^2^ is 73.7% less likely to be anemic when compared to BMI ranges from 18.5 to 24.9 kg/m^2^ or normal BMI. Overweight was inversely associated with the prevalence of anemia. This is consistent with a study conducted in Andhra Pradesh, India, in which overweight and obese women have lower prevalence ratios for anemia [43], and a study conducted in Jiangsu Province, China, among adult women [44]. This protective effect can be related to the nature of the diet and lifestyle of the individual. That is because BMI is directly related to the nutritional status. This suggests that underweight is related to the nutritional deficiency and results in the reduced bioavailability of essential nutrients and finally leads to anemia. The prevalence of anemia showed a significant difference between underweight and overweight that underweight populations are at risk of developing anemia [45]. A study conducted in Bangladesh also reported that being undernourished was more likely to be anemic compared to normal overweight and obese nonpregnant reproductive-aged women [30].

### 5.1. Limitations and Strength of the Study

The strength of the study was its study population; that is, it was community-based study and assessment of associated factors which may be important to plan intervention methods to decrease the prevalence of anemia. When we come to the limitations, being cross-sectional was the limitation for this study because it is difficult to show cause–effect relationship by the cross-sectional study. The other limitation is that we have used only Hb for anemia definition. We did not assess other hematologic parameters, such as iron, vitamin B12, folate, CBC, and the RBC morphology. In addition to this, lack of data on malaria and inflammation were limitations of the study. The other limitation of this study was that we only used wet mount examination for stool examination; as a result, we could not quantify and measure the severity of parasite.

## 6. Conclusion

The prevalence of anemia in the study area was a moderate public health problem. In this study, low educational status, BMI above 25 kg/m^2^, food insecurity, and age category of 36–49 years of the study participants were significantly associated with the prevalence of anemia. Therefore, it is better to design appropriate interventional strategies to reduce RAW anemia. These include information, education, and communication activities focused on RAW with no formal education, life-cycle-focused food security rather than targeted to only infants and young children or pregnant women and RAW, especially older-age groups need to visit health facilities and check themselves regularly for anemia. Further longitudinal studies with a large sample size including the assessment of all red blood cell indices, red cell morphology, serum micronutrient level, parasitic investigations with advanced methods, and subclinical infections need to be conducted in order to identify the cause–effect relationship of anemia with its contributing factors.

## Figures and Tables

**Table 1:** Sociodemographic, socioeconomic, and reproductive health-related characteristics of the study participants, from February to April 2018.

Variables		Frequency (*n*)	Percent
Age category	15–24	169	47.1
	25–35	130	36.2
	36–49	60	16.7

Marital status	Married	140	39.0
	Single	157	43.7
	Divorced	54	15.0
	Widowed	8	2.2

Educational status	No formal education	87	24.2
	Elementary school	62	17.3
	Secondary school and above	210	58.5

Religion	Orthodox	334	93.0
	Muslim	25	7.0

Household size	≤2	147	40.9
	3-4	145	40.4
	≥5	67	18.7

Occupation	Employee	31	8.6
	Housewife	122	34.0
	Student	128	35.7
	Merchant	31	8.6
	^*∗*^Others	47	13.1

Wealth index	Poor	150	41.8
	Middle	91	25.3
	Rich	118	32.9

Experience of pregnancy	Yes	171	47.6
	No	188	52.4

Number of pregnancies (*n* = 171)	1-2	98	27.3
	>2	73	20.3

Time gap between each birth (*n* = 130)	≤2	31	23.8
	>2	99	76.2

Bleeding history	Yes	26	7.2
	No	333	92.8

Oral contraceptive usage	Yes	26	7.2
	No	333	92.8

Menstruation cycle	Once per month	323	90.0
	More than once per month	36	10.0

Length of day blood flow in each menstruation	1–5	299	83.3
	>5	60	16.7

^*∗*^Others = daily laborers and job seekers.

**Table 2:** Prevalence and associated factors of anemia among RAW in Sayint Adjibar town, February to April 2018.

Variables	Variable categories	Anemia status			
		Nonanemic	Anemic	COR (95% CI)	AOR (95% CI)
Age	15–24	136 (80.5%)	33 (19.5%)	1.00	1.00
	25–35	103 (79.2%)	27 (20.8%)	1.08 (0.61–1.91)	0.83 (0.41–1.65)
	36–49	33 (55.0%)	27 (45.0%)	3.37 (1.79–6.36)	2.64 (1.05–6.60)

Marital status	Married	110 (78.6%)	30 (21.4%)	1.00	
	Single	123 (78.3%)	34 (21.7%)	1.01 (0.58–1.76)	
	Divorced and widowed	34 (63.0%)	20 (37.0%)	2.16 (1.12–4.16)	

Educational status	No formal education	50 (57.5%)	37 (42.5%)	3.35 (1.93–5.81)	2.28 (1.06–4.92)
	Elementary school	50 (80.6%)	12 (19.4%)	1.09 (0.53–2.24)	0.82 (0.34–1.95)
	Secondary school and above	172 (81.9%)	38 (18.1%)	1.00	1.00

Occupation	Employee	25 (80.6%)	6 (19.4%)	1.50 (0.844–2.648)	
	Housewife	86 (70.5%)	36 (29.5%)	1.00	
	Student	100 (78.1%)	28 (21.9%)	0.86 (0.320–2.294)	
	Merchant	23 (74.2%)	8 (25.8%)	1.24 (0.501–3.077)	
	^*∗*^Others	38 (80.9%)	9 (19.1%)	0.85 (0.366–1.957)	

BMI	<18.5	48 (80.0%)	12 (20.0%)	0.71 (0.36–1.41)	0.92 (0.45–1.92)
	18.5–24.9	201 (73.9%)	71 (26.1%)	1.00	1.00
	≥25	23 (85.2%)	4 (14.8%)	0.50 (0.17–1.47)	0.27 (0.08–0.87)

Presence of intestinal parasites	Yes	158 (78.2%)	44 (21.8%)	1.354 (0.834–2.199)	
	No	114 (72.6%)	43 (27.4%)	1.00	

HHFS	Secured food	236 (79.2%)	62 (20.8%)	1.00	1.00
	Insecure food	36 (59.0%)	25 (41.0%)	2.64 (1.48–4.73)	1.92 (1.01–3.65)

Bleeding history	Yes	16 (61.5%)	10 (38.5%)	2.08 (0.91–4.77)	
	No	256 (76.9%)	77 (23.1%)	1.00	

AOR = adjusted odds ratio, BMI = body mass index, CI = confidence interval, COR = crude odds ratio, and HHFS = household food security. ^*∗*^Others = daily laborers and job seekers.

## Data Availability

All data supporting the findings and conclusion are presented in the manuscript. Data used to support the findings of this study are also available from the corresponding author upon reasonable request.

## Abbreviations

AOR: Adjusted odds ratio
BMI: Body mass index
CI: Confidence interval
COR: Crude odds ratio
EDHS: Ethiopian Demographic and Health Survey
Hb: Hemoglobin
RAW: Reproductive-aged women
RBC: Red blood cells
WHO: World Health Organization.
